# Adipocyte-derived PGE_2_ is required for intermittent fasting–induced Treg proliferation and improvement of insulin sensitivity

**DOI:** 10.1172/jci.insight.153755

**Published:** 2022-03-08

**Authors:** Chunqing Wang, Xing Zhang, Liping Luo, Yan Luo, Xin Yang, Xiaofeng Ding, Lu Wang, Huyen Le, Lily Elizabeth R. Feldman, Xuebo Men, Cen Yan, Wendong Huang, Yingmei Feng, Feng Liu, Xuexian O. Yang, Meilian Liu

**Affiliations:** 1 Department of Biochemistry and Molecular Biology, University of New Mexico Health Sciences Center, Albuquerque, New Mexico, USA.; 2 Baodi Clinical College of Tian Jin Medical University, Tianjin, China.; 3 Beijing Youan Hospital, Capital Medical University, Beijing, China.; 4 Department of Diabetes Complications & Metabolism Research, City of Hope, Duarte, California, USA.; 5 Metabolic Syndrome Research Center, Second Xiangya Hospital, Central South University, Changsha, China.; 6 Department of Molecular Genetics and Microbiology and; 7 Autophagy Inflammation and Metabolism Center for Biomedical Research Excellence, University of New Mexico Health Sciences Center, Albuquerque, New Mexico, USA.

**Keywords:** Metabolism, Adipose tissue, Innate immunity

## Abstract

The intermittent fasting (IF) diet has profound benefits for diabetes prevention. However, the precise mechanisms underlying IF’s beneficial effects remain poorly defined. Here, we show that the expression levels of cyclooxygenase-2 (COX-2), an enzyme that produces prostaglandins, are suppressed in white adipose tissue (WAT) of obese humans. In addition, the expression of COX-2 in WAT is markedly upregulated by IF in obese mice. Adipocyte-specific depletion of COX-2 led to reduced fractions of CD4^+^Foxp3^+^ Tregs and a substantial decrease in the frequency of CD206^+^ macrophages, an increase in the abundance of γδT cells in WAT under normal chow diet conditions, and attenuation of IF-induced antiinflammatory and insulin-sensitizing effects, despite a similar antiobesity effect in obese mice. Mechanistically, adipocyte-derived prostaglandin E_2_ (PGE_2_) promoted Treg proliferation through the CaMKII pathway in vitro and rescued Treg populations in adipose tissue in COX-2–deficient mice. Ultimately, inactivation of Tregs by neutralizing anti-CD25 diminished IF-elicited antiinflammatory and insulin-sensitizing effects, and PGE_2_ restored the beneficial effects of IF in COX-2–KO mice. Collectively, our study reveals that adipocyte COX-2 is a key regulator of Treg proliferation and that adipocyte-derived PGE_2_ is essential for IF-elicited type 2 immune response and metabolic benefits.

## Graphical abstract

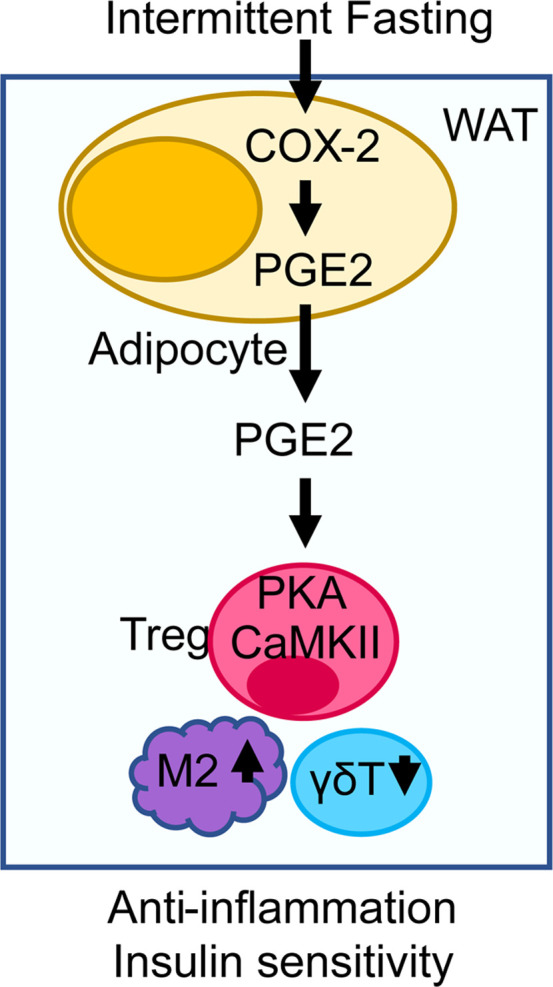

## Introduction

Obesity, a disorder characterized by excess adiposity, has become a primary cause of insulin resistance, type 2 diabetes, and cardiovascular diseases. Adipose tissue (AT) expansion in obesity is mediated by increased volume of preexisting adipocytes (hypertrophy), generation of new small adipocytes (hyperplasia), or both. Pathological expansion of AT is accompanied by adipose hypoxia, angiogenesis, remodeling, and inflammation, thereby causing systemic insulin resistance and type 2 diabetes (1, 2). Intermittent fasting (IF), an eating pattern that involves periods of normal eating followed by restriction (fasting), has gained attention as an effective approach to improve obesity-induced metabolic dysfunctions such as insulin resistance and type 2 diabetes (3–5). However, the mechanisms underlying the beneficial effects of IF on metabolic homeostasis are still poorly understood. This knowledge gap must be addressed to produce translational targets for antiobesity treatments in humans.

CD4^+^Foxp3^+^ Tregs are enriched in AT and are inversely correlated with obesity (6–8). AT Tregs exert antiinflammatory effects, and the reduction of AT Treg populations is crucially involved in the development of obesity-induced inflammation and substantial insulin resistance (6, 9, 10). Distinct from those Tregs in lymphoid and nonlymphoid tissue, AT Tregs are characterized by a distinguishable T cell receptor repertoire, chemokine profile, and chemokine receptors (6). Several intrinsic pathways, including PPARγ, IRF4, BATF, Stat3, and Stat6, play important roles in regulating development and activation of AT Tregs (9, 11–13). MHCII in adipocytes is a critical determinant of the obesity-induced, adipose T cell subset switch (14, 15). In addition, cold stress has been demonstrated to induce activation of Tregs as well as development of beige adipocytes in AT (13). However, little is known about whether caloric restriction, such as IF, improves metabolic homeostasis by regulating Treg proliferation and activation.

Cyclooxygenase (COX), a rate-limiting enzyme responsible for the biosynthesis of prostaglandins (PGs), exists in 2 isoforms: COX-1, the constitutive form encoded by *Ptgs1*, and COX-2, the inducible form encoded by *Ptgs2* (16). COX-2 oxygenates arachidonic acid and converts it into a number of PGs, including PGD_2_, PGE_2_, PGF_2α_, and prostacyclin (PGI_2_), all of which exert diverse hormone-like effects via autocrine or paracrine mechanisms (16). The accumulated evidence shows that PGs modulate adipogenesis by acting as an agonist or analog of PPARγ (17). It has also been suggested that the COX-2/PG axis plays a critical role in regulating AT inflammation and obesity-induced insulin resistance (18–23). In addition, the COX-2/PG pathway is involved in thermogenic programming and WAT browning, although whether COX-2 is induced by cold and is correlated with obesity are still controversial (18, 24–27). On the other hand, while COX-2 expression is known to be induced by activation of lipolysis, a pathway that is stimulated by IF (21), whether adipocyte COX-2 is altered by IF and mediates IF-driven beneficial effects is unclear.

Here, we show that adipose COX-2 is suppressed by obesity in humans, and the decreased levels of COX-2 were restored by alternate-day fasting in obese rodents. Adipose-specific depletion of COX-2 leads to a significant decrease in the fractions of Tregs and CD206^+^ macrophages and increased abundance of γδT cells in AT, overall suppressing AT type 2 inflammation under normal chow diet (NCD) conditions. COX-2 deficiency resulted in adipocyte-suppressed, IF-induced Treg activation and insulin sensitivity via PGE_2_-dependent mechanisms. In this study, we report that adipocyte-derived PGE_2_ senses fasting and refeeding and mediates IF-elicited metabolic fitness. These findings provide a detailed and relevant basis for continued translational research regarding PGE_2_ as a human anti-diabetes treatment.

## Results

### COX-2 expression is suppressed by obesity, and this suppression is restored by IF in AT.

**Figure 1:**
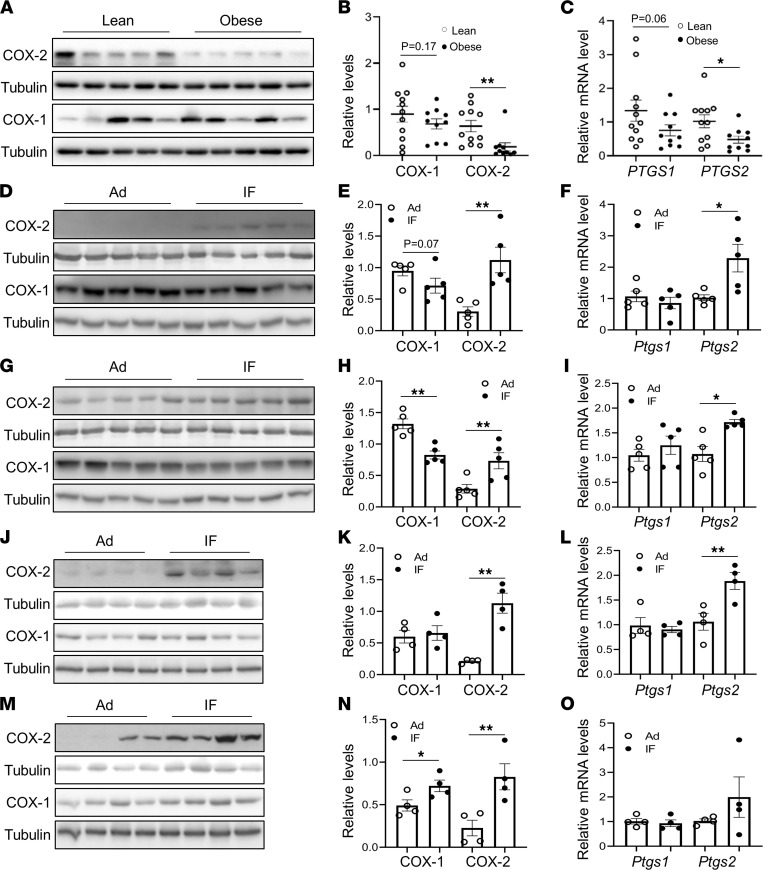
COX-2 expression is suppressed by obesity and is induced by IF in AT. The expression levels of COX-2 but not COX-1 in protein (**A **and** B**) and mRNA (**C**) were suppressed by obesity in visceral fat of human participants, compared with that of lean controls, despite a decrease in mRNA of PTGS2, a gene that encodes human COX-2. *PTGS1* is a gene encoding human COX-1. Tubulin was used as the loading control. The expression levels of COX-2 but not COX-1 in protein (**D** and **E**) and mRNA (**F**) were markedly induced by IF in eWAT of mice with diet-induced obesity. The protein (**G** and **H**) and mRNA (**I**) levels of COX-2 in iWAT were also induced by IF despite the suppression of COX-1 in protein in obese mice. The upregulation of COX-2 expression by IF was also observed in eWAT (**J–L**) and iWAT (**M** and **N**) of NCD mice, although no significant effect was observed on the mRNA levels of both *Ptgs2,* a gene that encodes mouse COX-2, and *Ptgs1,* a gene that encodes mouse COX-1, in iWAT (**O**). We fed 6-week-old male mice a 45% HFD or NCD for 8 weeks, which was followed by Ad or IF for 30 days. All data in this figure were analyzed by *t* test and are presented as the mean ± SEM. *P < 0.05; **P < 0.01.

Adipose COX-2 plays a vital role in regulating inflammation and energy expenditure by controlling synthesis of PGs (18, 20–23). To investigate whether obesity affects the expression levels of adipose COX-2 in humans, we collected visceral fat samples from patients with obesity or overweight with BMI greater than 27 kg/m^2^ and lean control participants with BMI less than 25 kg/m^2^. Consistent with our previous finding that COX-2 is downregulated in white adipose tissue (WAT) of obese mice (26), we found that the protein and mRNA levels of COX-2 were significantly reduced in visceral fat of human participants with obesity (Figure 1, A–C). Interestingly, 15-cycle IF restored the protein and mRNA levels of COX-2 but not of COX-1 in epididymal WAT (eWAT) and inguinal WAT (iWAT) of mice with diet-induced obesity (Figure 1, D–I). Consistent with this, the expression levels of COX-2 but not COX-1 in eWAT were also significantly upregulated by 3-cycle IF (Supplemental Figure 1, A and B; supplemental material available online with this article; https://doi.org/10.1172/jci.insight.153755DS1) and 15-cycle IF (Figure 1, J–L) in the mice fed a NCD, although the induction of COX-2 in iWAT was only observed in 15-cycle IF mice (Figure 1, M–O) but not in 3-cycle IF mice (Supplemental Figure 1, C and D). The results suggest a negative correlation of adipose COX-2 with obesity in humans and that adipose COX-2 may be involved in IF-driven metabolic benefits.

### Adipocyte-specific depletion of COX-2 attenuates PG production and promotes age-related adipogenesis.

**Figure 2:**
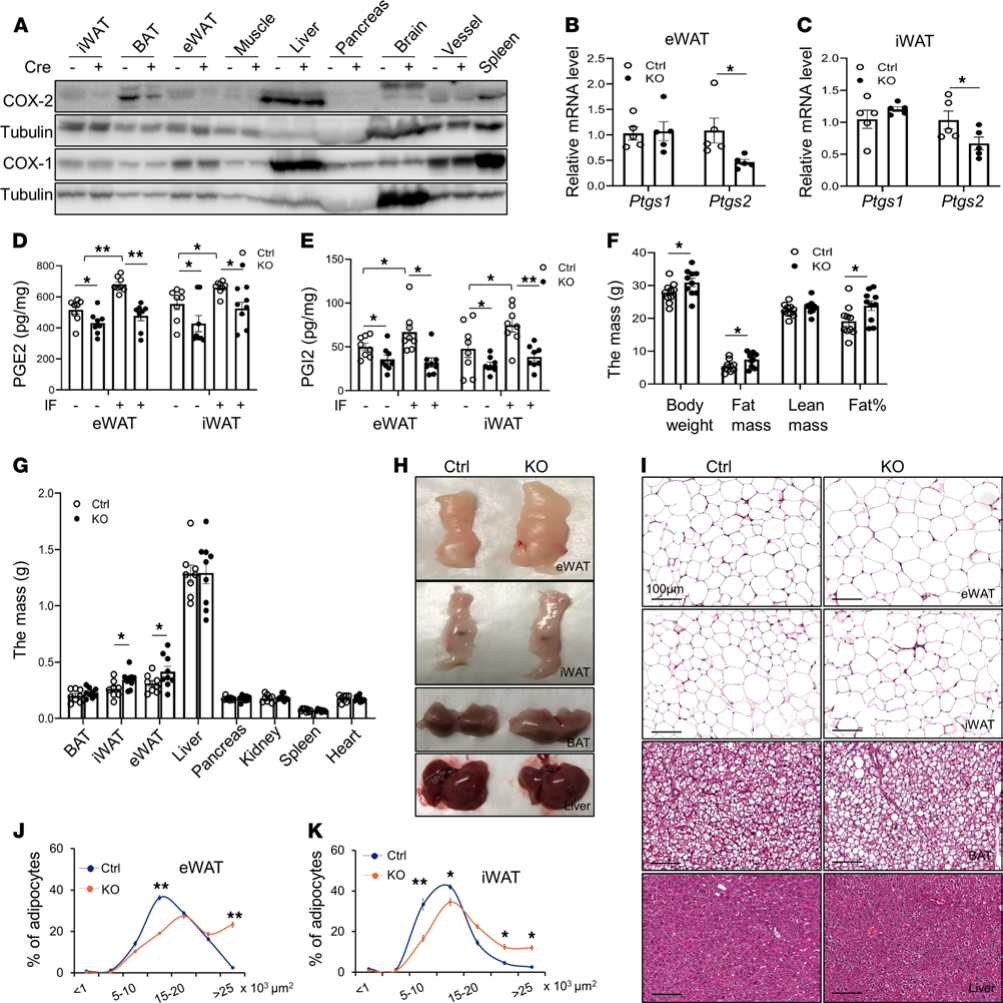
Adipocyte-specific depletion of COX-2 suppressed IF-induced PG production and increased adipocyte development. (**A–E**) Three-month-old male COX-2–KO and control (Ctrl) mice were used. (**A**) COX-2 was highly enriched in AT compared with other tissue or organs, and adipocyte-specific depletion of COX-2 markedly downregulated COX-2 but not COX-1 protein levels in eWAT, iWAT, and BAT, with little effect on muscle, liver, pancreas, brain, or blood vessels. (**B** and **C**) The mRNA levels of *Ptgs2* but not *Ptgs1* were notably decreased by COX-2 depletion in eWAT and iWAT. The basal and IF secretion levels of PGE_2_ (**D**) and PGI_2_ (**E**) in eWAT and iWAT were significantly decreased in COX-2–KO mice compared with Ctrls. The tissue samples of eWAT and iWAT were minced and incubated in medium for 8 hours. The medium was collected and the levels of PGE_2_ and PGI_2_ were determined by an ELISA kit accordingly. (**F–K**) Six-month-old male COX-2–KO and Ctrl mice were used for these studies. (**F**) COX-2 deficiency promoted adipocyte development. The lean mass, fat mass, total mass, and fat percentage were measured using dual-energy X-ray absorptiometry scanning. (**G**) The mass of epididymal and inguinal fat pads, but not of brown fat, was increased in COX-2–KO mice. The organs were weighed after mice were euthanized. (**H**) Representative images of eWAT, iWAT, BAT, and liver in COX-2–KO and Ctrl mice. (**I**) H&E staining of eWAT, iWAT, BAT, and liver in COX-2–KO and Ctrl mice. (**J** and **K**) The fat cells’ size was enlarged by COX-2 deficiency in eWAT and iWAT. (**B–G**, **J**, and **K**) Data are presented as the mean ± SEM. (**B**, **C**, **F**, **G**, **J**, and **K**) Data were analyzed via *t* test. (**D** and **E**) ANOVA was used for statistical analysis. *P < 0.05; **P < 0.01.

To characterize the physiological role of adipocyte COX-2 in regulating adipose and systemic metabolism, we generated adipocyte-specific COX-2–KO mice by crossing COX-2–floxed mice and adiponectin-cre mice. The results showed that both protein and mRNA levels of COX-2 but not COX-1 in eWAT and iWAT, and not in muscle, liver, pancreas, or brain tissue, were markedly downregulated by adipocyte-specific depletion of COX-2 (Figure 2, A–C). A similar suppressing effect of COX-2 depletion on COX-2 was observed in adipocytes (Supplemental Figure 1E). COX-2 deficiency in adipocytes suppressed the basal and IF-induced secretion of PGE2 in eWAT and iWAT (Figure 2D and Supplemental Figure 1, F and G). COX-2 deficiency also decreased the secretion levels of PGI_2_ in WAT under basal and IF conditions (Figure 2E). PGD_2_ levels were not significantly affected by COX-2 deficiency and IF even with a slight increase in KO eWAT (Supplemental Figure 1H).

Contrary to previous findings that global depletion of COX-2 led to fat loss (19), the COX-2–deficient mice displayed a slight increase in fat mass and total body mass under NCD conditions at the age of 3 months (Supplemental Figure 1I) but to a greater extent in 6-month-old mice (Figure 2, F–H). The increased fat mass occurred in eWAT and iWAT with a slight increase in brown adipose tissue (BAT) of 6-month-old COX-2–KO mice compared with control littermates (Figure 2, F–I). Moreover, adipocyte size in iWAT and eWAT was increased by adipocyte COX-2 deficiency (Figure 2, I–K). Consistent with this, COX-2–deficient mice exhibited slightly reduced glucose and insulin tolerance at 6 months of age but not at 3 months (Supplemental Figure 1, J–M). These data suggest that adipocyte COX-2 maybe a key regulator of WAT adipogenesis and function.

### COX-2 deficiency in adipocytes suppresses Treg proliferation and type 2 inflammation in AT.

**Figure 3:**
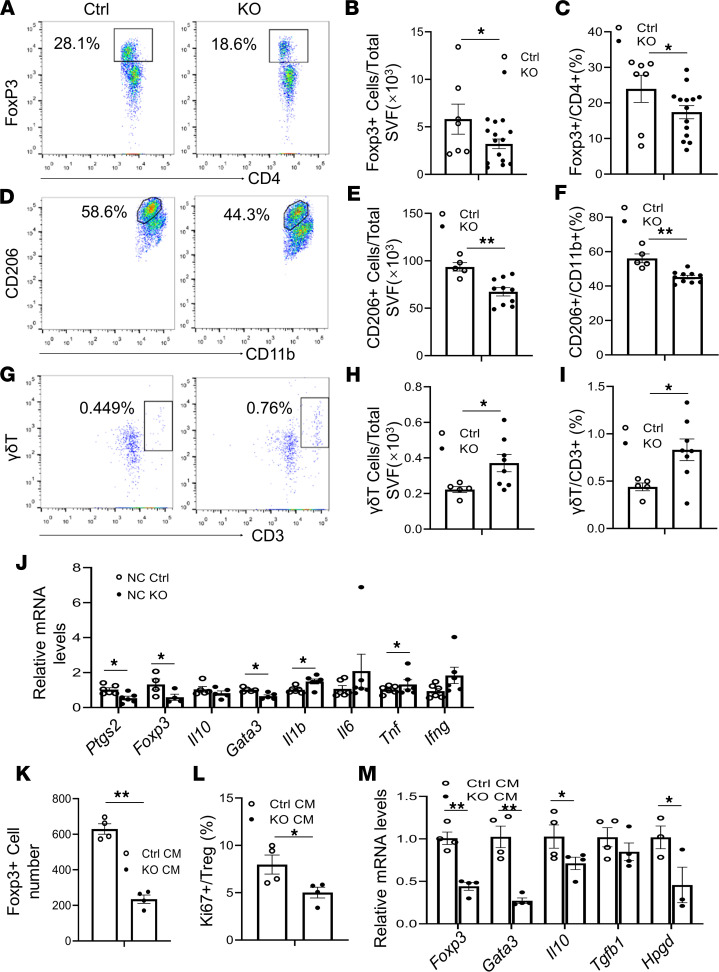
Deficiency of COX-2 in adipocytes reduces Treg frequency and type 2 immune response in AT. (**A–I**) The eWAT samples were collected from 6**-**month-old male COX-2–KO and control (Ctrl) mice (*n* = 5–14/group); the stromal vascular fraction of AT was used for flow cytometry analysis. (**A**) The fraction of resident CD4^+^Foxp3^+^ cells was decreased in eWAT of COX-2–KO mice compared with that of Ctrl mice. (**B** and **C**) The percentage of CD4^+^ Foxp3^+^ cells in total CD4^+^ cells was decreased in eWAT of COX-2–KO mice compared with that of Ctrl mice. COX-2–KO mice had reduced a Siglec-5^–^CD11b^+^CD206^+^ population (**D** and **E**) and decreased proportion of CD11b^+^CD206^+^ in CD11b^+^ cells (**F**) in eWAT. COX-2 deficiency led to an increase in the γδT^+^CD3^+^ cell population (**G** and **H**) and in the proportion of γδT^+^CD3^+^ cells in total CD3^+^ cells (**I**) in eWAT. (**J**) COX-2–KO downregulated mRNA levels of Foxp3, GATA-3 as well as COX-2 in eWAT while upregulating levels of IL-1β and IFN-γ in eWAT despite no significant effect on IL-10, IL-6, and TNF-α. (**K** and **L**) The culture medium (CM) from primary COX-2–KO adipocytes decreased the fraction of CD4^+^Foxp3^+^ cells and suppressed the percentage of Ki67^+^ Tregs in total Tregs compared with Ctrl medium. AT Tregs were isolated from AT and treated with the CM from COX-2–KO and Ctrl primary adipocytes for 24 hours. *n* = 4/group. (**M**) The medium from primary COX-2–KO adipocytes decreased mRNA levels of *Foxp3*, *Gata3*, *Il10*, and *Hpgd* without significant effects on *Tgfb1* in AT Tregs. (**B**, **C**, **E**, **F**, and **H–M**) The *t* test was used to analyze data for these studies. All data are presented as mean ± SEM. * *P* < 0.05; **P < 0.01.

To investigate the role of COX-2 in regulating AT inflammation, we performed flow cytometry analysis to examine whether deficiency of COX-2 in adipocytes alters adipose immune compartment at age 6 months, using the strategy described in Supplemental Figure 2A. Despite no significant decrease in CD4^+^, CD3^+^, CD8^+^, or CD45^+^ T cells (Supplemental Figure 2, B–E), COX-2 deficiency decreased the frequency of CD4^+^Foxp3^+^ cells (Tregs) and the proportion of Tregs in CD4^+^ cells in eWAT (Figure 3, A–C), suggesting the antiinflammatory effect of COX-2 in adipocytes. Moreover, both the fraction of Siglec-5^–^CD11b^+^CD206^+^ cells and the proportion of CD206^+^ macrophages in total macrophages were suppressed by COX-2 deficiency (Figure 3, D–F), accompanied with an increased proportion of M1 (Siglec-5^–^CD11b^+^CD206^–^) in total macrophage, decreased total macrophages, and reduced fraction of eosinophils (Siglec-5^+^CD11b^+^) (Supplemental Figure 2, F–H). In addition, the fraction of γδT cells was increased by COX-2 deficiency in eWAT under NCD conditions (Figure 3, G–I).

Along this line, the mRNA levels of Treg markers, including *Foxp3* and *Gata3*, were significantly decreased in COX-2–KO mice, while inflammatory factors *Ifng* and *Il1b* were upregulated with no significant effect on *Il10*, *Il6*, and *Tnf* (Figure 3J). In agreement with the antiinflammatory property of COX-2 in adipocytes, the medium from COX-2–KO adipocytes did not maintain AT Treg proliferation, as indicated by the number of Foxp3^+^CD4^+^ cells, the proportion of Ki67^+^ Tregs in total Tregs, and expression levels of *Foxp3*, *Gata3*, *Hpgd*, and *Il10*, but not *Tgfb1* (Figure 3, K–M).

### COX-2 in adipocytes is required for IF-driven metabolic improvement in AT inflammation and insulin sensitivity.

**Figure 4:**
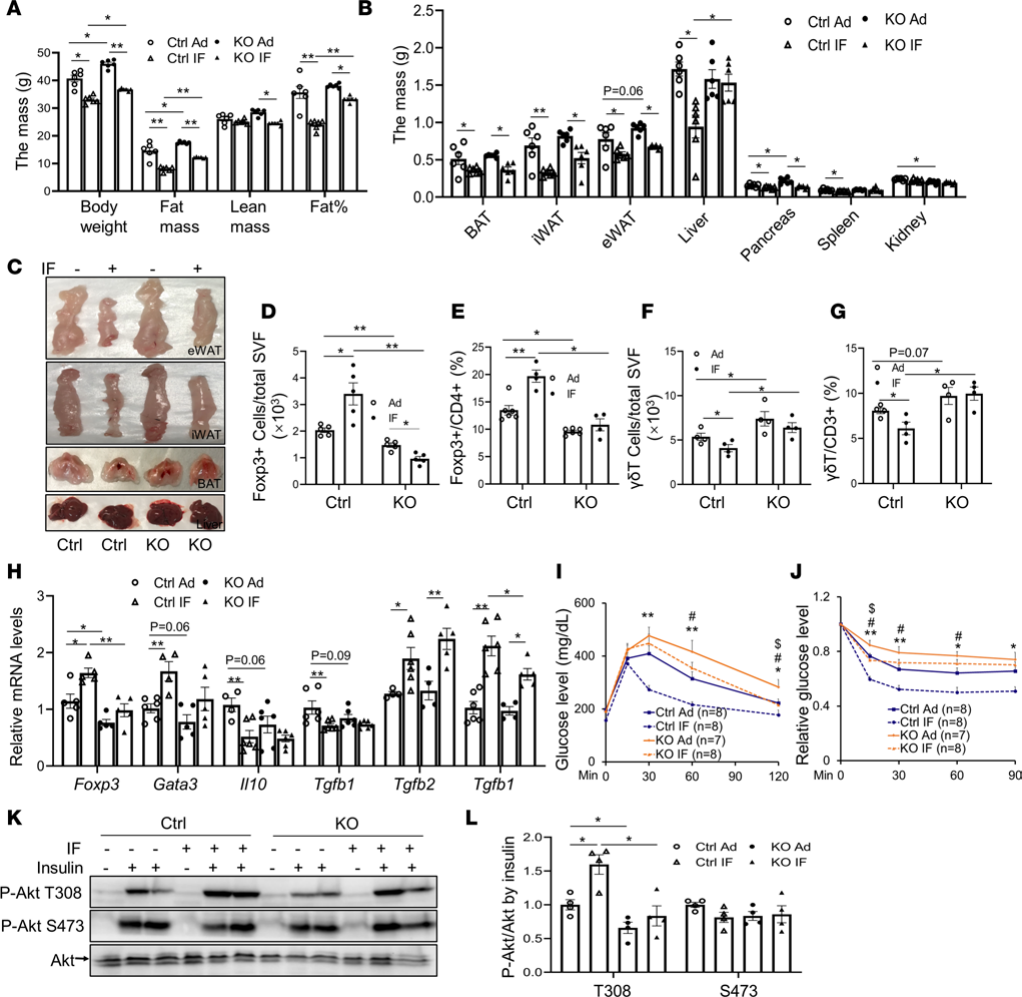
COX-2 deficiency suppressed IF-induced Treg proliferation and improvement of insulin resistance. A HFD was fed to 6-week-old male COX-2–KO and control (Ctrl) mice for 8 weeks followed by IF (*n* = 6–8/group) for 30 days. (**A**) IF led to a 32.6% loss of fat mass and a 18.7% loss of body mass but not lean mass in Ctrl mice, and the antiobesity effect was not significantly affected by COX-2 deficiency. (**B**) IF-induced mass loss in eWAT and BAT was little affected, whereas the effects on iWAT and liver were suppressed in COX-2–KO mice compared with Ctrl mice. (**C**) Representative images of eWAT, iWAT, BAT, and liver in HFD-fed COX-2–KO and Ctrl mice before and after IF. COX-2 deficiency alleviated IF-induced increase in the Treg fraction (**D**) and the proportion of Tregs in CD4^+^ cells (**E**); suppressed the inhibitory effects of IF on the γδT cell fraction (**F**) and the proportion of γδT cells in CD3^+^ cells (**G**); and diminished the inducing effect of IF on mRNA levels of Foxp3, GATA3, and TGFβ3 with little effect on IL-10, TGFβ1, and TGFβ2 (**H**) in eWAT. (**I**) COX-2 deficiency diminished IF-improved glucose tolerance. (**J**) COX-2 deficiency diminished IF-improved insulin tolerance. (**K** and **L**) Insulin-stimulated phosphorylation of Akt at Thr308 (T308) and Ser473 (S473) in the liver of COX-2–KO and Ctrl mice treated with or without IF. *n* = 4/group. *P < 0.05 and **P < 0.01 for Ad vs. IF in Ctrl mice; ^#^*P* < 0.05 for Ctrl vs. KO mice with Ad diet; ^$^*P* < 0.05 for Ad vs. IF in KO mice. ANOVA was used to analyze all the data in this figure. (**A**, **B**, and **D–H**) Data are presented as mean ± SEM. * *P* < 0.05; **P < 0.01.

The marked induction of COX-2 expression by IF in AT spurred us to investigate if COX-2 in adipocytes mediates IF-elicited metabolic benefits (Figure 1, D–I). COX-2–KO mice and control littermates (6 weeks old) were fed an HFD for 8 weeks and then underwent alternate-day fasting (24-hour fasting followed by alternating 24-hour periods with free access to food) for 15 cycles. We found that IF led to a robust decrease in fat mass, body mass, and fat cell size in WAT, as well as smaller lipid droplets in BAT and liver tissue in HFD-fed control mice (Figure 4, A–C, and Supplemental Figure 3, A–C), and the total food intake for each cycle was similar among the ad libitum (Ad), IF, control, and KO groups (Supplemental Figure 3D). However, although COX-2–KO mice displayed an increase in body mass, fat mass, and fat cell size, the antiobesity effect of IF was retained in COX-2–KO mice (Figure 4, A–C, and Supplemental Figure 3, A–C). The suppressing effects of IF on fat percentage, iWAT mass, and fatty liver were attenuated by COX-2 deficiency (Figure 4, A and B, and Supplemental Figure 3, A and E). On the other hand, IF restored HFD-decreased frequency of Tregs and mRNA levels of *Foxp3*, *Gata3*, *Tgfb2*, and *Tgfb3* accompanied by a decreased γδT cell fraction (Figure 4, D–H, and Supplemental Figure 3, F and G). In agreement with this, insulin-stimulated Akt phosphorylation at Thr308 in liver and eWAT, as well as glucose and insulin tolerance, were improved by IF in HFD-fed mice (Figure 4, I–L, and Supplemental Figure 3H). However, the improvement in AT inflammation, insulin action, and insulin sensitivity were suppressed in COX-2–KO mice concurrently with decreased fractions of Tregs and CD206^+^ macrophages and an increased γδT cell population (Figure 4, D–L, and Supplemental Figure 3, F–H). Depleting COX-2 in adipocytes suppressed IF-induced UCP1 and adiponectin expression and upregulated PPARγ expression levels in iWAT (Supplemental Figure 3I).

We asked if COX-2 induces thermogenic markers through VEGF and FGF21 pathways, given their role in fasting adaptive response and subsequent WAT browning (28–30). COX-2 deficiency had little effect on basal and IF-induced *Vegf* expression and *Fgf21* transcription in iWAT (Supplemental Figure 3J). IF, however, suppressed transcription of proinflammatory genes *Ifng* and histocompatibility 2-class II antigen E beta (*H2-Eb1*) that have been shown to suppress AT Treg activation (14), which is independent of COX-2 in eWAT (Supplemental Figure 3J). Our results suggest that adipocyte COX-2 partially mediates IF-induced Treg proliferation and improved AT inflammation and insulin sensitivity, which may be obesity independent (Figure 4).

### Adoptive transfer of Treg restores the impaired antiinflammatory response and insulin sensitivity in COX-2–deficient mice.

**Figure 5:**
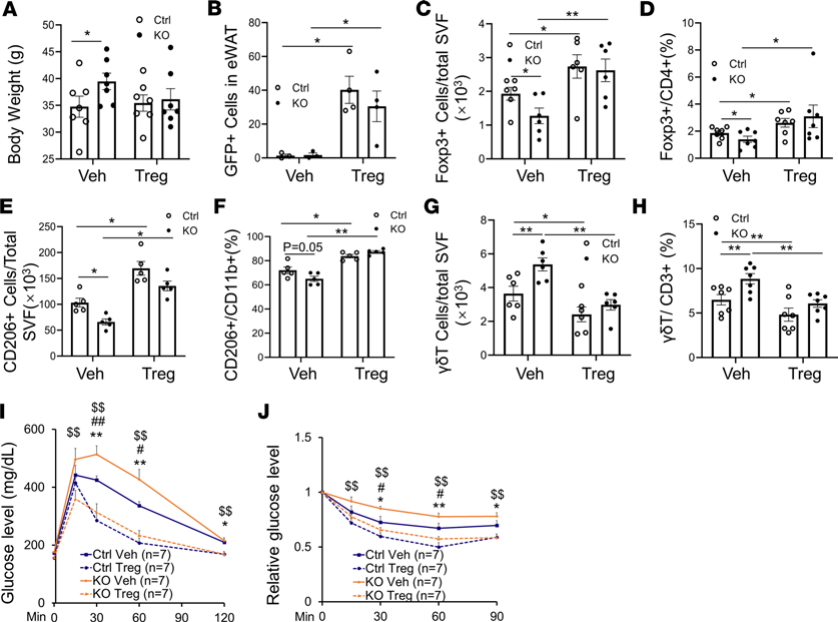
Adoptive transfer of Tregs reverses COX-2–KO–caused AT inflammation and insulin resistance. For the following studies, mouse GFP^+^CD4^+^ T cells were isolated from lymph nodes and spleens of Foxp3-eGFP mice, and IP injection of CD4^+^GFP^+^ T cells which were positive Treg cells and CD4^+^GFP^-^ as negative control cells to 8 weeks HFD-fed COX-2–KO and control (Ctrl) mice. (**A**) There was little effect of adoptive transfer on the BW in COX-2–KO and control mice 2 weeks post transfer. Flow cytometry analysis of CD4^+^GFP^+^ cells (**B**), CD4^+^Foxp3^+^ Treg cells (**C**) and the proportion of Foxp3^+^ Treg in CD4^+^ cells (**D**) in eWAT showed the successful transfer of Tregs in AT. Adoptive transfer of Tregs increased CD11b^+^CD206^+^ cell fraction (**E**) and the proportion of CD11b^+^CD206^+^ in CD11b^+^ cells (**F**), while suppressed γδT^+^CD3^+^ cell population (**G**) and the proportion of γδT^+^CD3^+^ cell in total CD3^+^ cells (**H**). Adoptive transfer of Treg cells rescued COX-2 deficiency-induced glucose (**I**) and insulin (**J**) intolerance. *P < 0.05 and **P < 0.01 Ctrl vehicle (Veh) vs. Ctrl Treg; ^#^*P* < 0.05 and ^##^*P* < 0.01 for Ctrl Veh vs. KO Veh; ^$$^*P* < 0.01 for KO Veh vs. KO Tregs; no significant difference was found between Ctrl Tregs and KO Tregs. (**A–H**) *n* = 4–7/group. (**B**) Representative data from 3 independent experiments are reported. ANOVA was used to analyze the data in this figure. Data are reported as mean ± SEM. *P < 0.05; **P < 0.01.

To determine whether the decrease in the Treg fraction mediates COX-2 deficiency–induced insulin resistance, we adoptively transferred CD4^+^Foxp3^+^ Tregs from Foxp3-eGFP mice into either COX-2–KO mice or control littermates. The CD4^+^GFP^+^ cells were selected from the isolated CD4^+^CD25^+^ cells and i.p. injected (1 × 10^5^ cells/mouse) into male COX-2–KO and control mice fed an HFD for 8 weeks. BW measurement and glucose and insulin tolerance tests were performed 14 days after the injection. Despite having no significant effects on BW (Figure 5A), adoptive transfer of Tregs increased the resident GFP^+^ cells and total CD4^+^Foxp3^+^ Tregs in eWAT (Figure 5, B and C). The increased fraction of CD4^+^GFP^+^ cells as well as total CD4^+^Fxop3^+^ cells in COX-2–KO WAT enhanced the γδT cell population and increased the CD206^+^ macrophage fraction (Figure 5, D–H). Consistent with this, adoptive transfer of Tregs restored insulin and glucose tolerance in COX-2–KO mice (Figure 5, I and J), indicating that resident Tregs are indispensable for the antiinflammatory and antidiabetic effects of COX-2 in adipocytes.

### Adipocyte COX-2 promotes Treg proliferation through PGE_2_.

**Figure 6:**
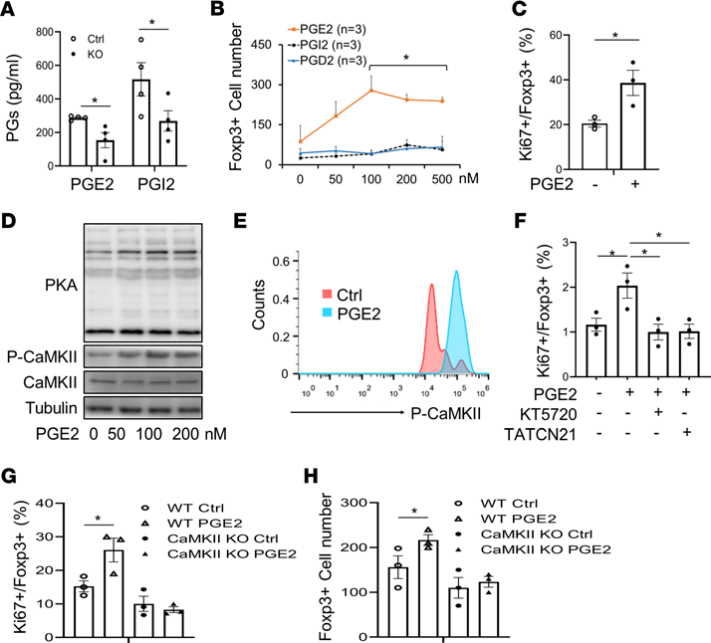
Adipocyte COX-2 promotes resident Treg proliferation through PGE_2_. (**A**) COX-2 deficiency suppressed the secretion levels of PGE_2_ and PGI_2_ in primary adipocytes. COX-2–KO and control (Ctrl) primary adipocytes were changed to fresh medium and cultured for 2 hours. Medium was collected and used to determine the levels of PGE_2_ and PGI_2_, using an ELISA kit. (**B**, **C**, **E**, and **F**) AT Tregs were isolated from AT for these studies. Treatment of PGE_2_ but not PGD_2_ and PGI_2_ increased the population of Foxp3^+^ Tregs in a dose-dependent manner (**B**), and treatment of 100 nM PGE_2_ induced proliferation of Tregs as indicated by the staining of Ki67 (**C**). Intracellular Ki67^+^ and Foxp3^+^ Tregs were determined by flow cytometry analysis. AT Tregs were treated with DMSO, PGD_2_, PGE_2_, or PGI_2_, with indicated doses for 24 hours. *P < 0.05 compared with the group without treatment. (**D**) Treatment of PGE_2_ stimulated activation of PKA and CaMKII in differentiated Tregs in a dose-dependent manner. CD4^+^-naive T cells were isolated from a single-cell suspension from lymph nodes and spleens and then differentiated into CD4^+^Foxp3^+^ Tregs. Differentiated Tregs were treated, or not, with PGE_2_ for 1 hour. Representative data from 3 independent experiments are reported. (**E**) PGE_2_ treatment stimulated phosphorylation of CaMKII in AT Tregs. *n* = 3/group. (**F**) Inhibiting PKA by 5 μM KT 5720 or inhibiting CaMKII by 5 μM TATCN21 suppressed PGE_2_-treatment–induced proliferation of AT Tregs. AT Tregs were treated with KT 5720 or TATCN21 for 1 hour, followed by co-treatment with PGE_2_ for 24 hours. *n* = 3/group. CaMKIIγ deficiency blocked PGE_2_-stimulated CD4^+^Foxp3^+^ Treg proliferation, indicated by Ki67 expression (**G**) and total Treg fraction (**H**). Primary Tregs were isolated from AT of CaMKIIγ-KO and WT mice and treated with 100 nM PGE_2_ for 24 hours, followed by flow cytometry analysis. (**A–C**) The *t* test was used for data analysis. (**F–H**) ANOVA was used for data analysis. Data are reported as mean ± SEM. *P < 0.05; **P < 0.01.

To dissect the role of adipocyte COX-2 in regulating resident Tregs, we analyzed the production of PGs in COX-2–KO and control primary adipocytes. COX-2 depletion suppressed the secretion of PGE_2_ and PGI_2_, but not PGD_2_, in adipocytes (Figure 6A and Supplemental Figure 3K), implying a PG-mediated paracrine mechanism. By treating AT Tregs with different doses of PGE_2_, PGI_2_, and PGD_2_, we found that PGE_2_, but not PGD_2_ or PGI_2_, significantly elevated the number of CD4^+^Fxop3^+^ Tregs in a dose-dependent manner (Figure 6B). Consistent with this, the treatment of 100 nM PGE_2_ elevated the proportion of Ki67-positive CD4^+^Fxop3^+^ Tregs in total Tregs (Figure 6C), suggesting that PGE_2_ may mediate the promoting effect of COX-2 on Treg proliferation.

Given that PGE_2_ activates PKA, ERK1/2, Akt, and CaMKII in a variety of cell types (31–34), we treated differentiated Tregs with different doses of PGE_2_ for 1 hour. PGE_2_ treatment stimulated phosphorylation of PKA substrate and CaMKII, but not ERK1/2 and Akt, in Tregs (Figure 6D and Supplemental Figure 3L). Treatment with PGE_2_ for 1 hour also stimulated phosphorylation of CaMKII in AT Tregs (Figure 6E). In addition, the promoting effect of PGE_2_ on cell proliferation was abolished by treating the cells with KT 5720, a specific inhibitor of PKA, or with TATCN21, a specific inhibitor of CaMKII, in AT Tregs (Figure 6F). Inhibiting PKA had a blocking effect on PGE_2_-induced stimulation of CaMKII in differentiated Tregs (Supplemental Figure 3M). In addition, CaMKII-KO Tregs isolated from AT exhibited lower response to PGE_2_-induced proliferation (Figure 6, G and H, and Supplemental Figure 3N). These findings suggest that PGE_2_ promotes Treg proliferation through the PKA-CaMKII axis.

### PGE_2_/Treg axis is required for IF-elicited insulin sensitivity.

**Figure 7:**
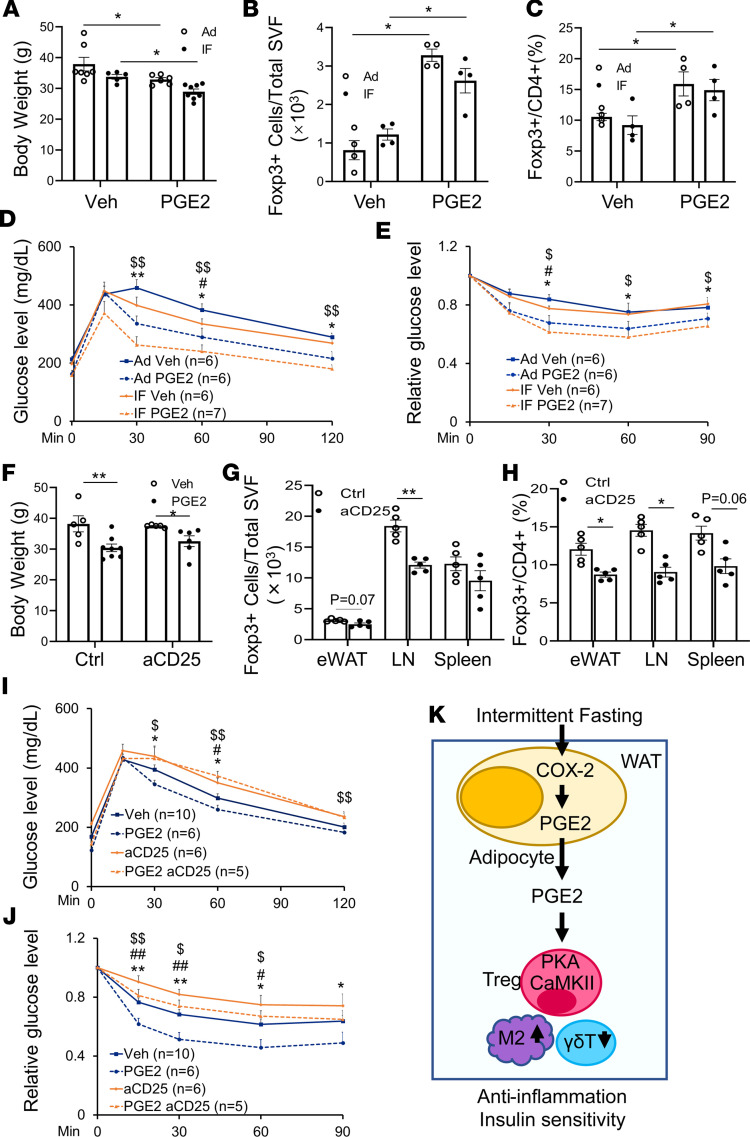
The PGE_2_/Treg axis is indispensable for the antiinflammatory and insulin-sensitizing effects of IF. HFD-fed COX-2–KO mice were fed on an IF schedule or Ad for 4 weeks. Two weeks after IF, mice were injected with PGE_2_ or vehicle (Veh) for 2 weeks. (**A**) PGE_2_ administration resulted in significantly decreased body mass of COX-2–KO mice under both IF and Ad conditions. *n* = 5–8/group. Treatment with PGE_2_ restored the AT Treg population (**B**) and the proportion of Tregs in CD4^+^ cells (**C**) in COX-2–KO mice under both IF and Ad conditions. *n* = 4/group. PGE_2_ administration improved glucose (**D**) and insulin (**E**) tolerance in COX-2–KO mice under both IF and Ad conditions. *P < 0.05 and **P < 0.01 for Veh vs. PGE_2_ with Ad diet; ^#^*P* < 0.05 for Ad vs. IF with Veh treatment; ^$^*P* < 0.05 and ^$$^*P* < 0.01 for IF Veh vs. IF PGE_2_. HFD-fed C57BL/6 mice were administered CD25 neutralizing antibody for 2 days, followed by PGE_2_ injection. (**F**) Blocking the Treg pathway had no significant effect on the antiobesity effect of PGE_2_, as indicated by the body mass. *n* = 5–8/group. (**G** and **H**) Neutralization of CD25 diminished the inducing effects of PGE_2_ on the AT Treg population. *n* = 5/group. Blocking the Treg pathway impaired basal and PGE_2_-increased glucose (**I**) and insulin (**J**) tolerance. *P < 0.05 and **P < 0.01 for Veh vs. PGE_2_; ^#^*P* < 0.05 and ^##^*P* < 0.01 for Ctrl vs. anti-CD25 (aCD25); ^$^*P* < 0.05 and ^$$^*P* < 0.01 for aCD25 vs. PGE_2_ aCD25. (**K**) Working model. ANOVA was used to analyze the data in this figure. All data are reported as mean ± SEM. LN, lymph node.

Given the mediatory role of PGE_2_ in adipocyte COX-2–driven activation of Tregs, we were motivated to investigate whether PGE_2_ mediates COX-2–elicited beneficial effects under IF conditions. We administered 50 μg/kg PGE_2_ i.p. for 2 weeks in COX-2–KO mice fed an IF diet (Figure 7, A–E). Although BW was slightly decreased as a result of IF in COX-2–KO mice, PGE_2_ administration led to significant body mass loss in COX-2–KO mice under both IF and Ad conditions (Figure 7A). In addition, while IF had no significant effect on AT Treg populations and glucose and insulin tolerance in COX-2–KO mice, the reduced beneficial effects were improved by PGE_2_ treatment (Figure 7, B–E), indicating that PGE_2_ is downstream of adipocyte COX-2 and plays a critical role in COX-2–mediated metabolic improvement.

We also asked if PGE_2_ improves insulin sensitivity through induction of Tregs. We injected CD25 neutralizing antibody for 14 days to block Treg function in HFD C57BL/6 mice during PGE_2_ treatment. Administration of PGE_2_ increased Treg fraction in eWAT, which was inactivated by anti-CD25 treatment (Figure 7, F–H). Moreover, suppressing Treg function abrogated PGE_2_-elicited AT Treg induction and insulin sensitivity (Figure 7, I and J). These results suggest that COX-2 in adipocytes, by regulating the COX-2/PGE2 axis, plays a critical role in IF-driven metabolic benefits. Our data also show that adipocyte COX-2 is required for IF to promote resident Treg proliferation and improve adipose inflammation and insulin sensitivity (Figure 7K).

## Discussion

Caloric restriction promotes weight loss, improves health, and extends lifespan in adult humans and rodents with obesity (35–38). IF is an alternative to continuous caloric restriction that cycles between fasting and nonfasting over a defined period and has been attracting increased attention as an effective approach for weight loss, WAT browning, and improvement of insulin sensitivity (3–5, 39–41). However, the precise mechanisms underlying the beneficial effects of IF are incompletely understood. Our study demonstrates that adipocyte COX-2 is induced by IF and mediates IF-induced CD4^+^Foxp3^+^ Treg proliferation and IF-improved inflammation in AT through PGE_2_ (Figure 7K). In addition, PGE_2_ promotes Treg proliferation via a CaMKII-dependent mechanism (Figure 7K). Our results reveal that adipocyte COX-2 is a promising therapeutic target for the treatment of obesity and its associated diseases.

COX-2 has been considered a key regulator of adipose inflammation and insulin resistance (20–23). However, the physiological signals that drive the activation of COX-2 in adipocytes remain largely unknown. COX-2 expression is restricted under basal conditions in adipocytes and is induced by various environmental stimuli such as adrenoceptor agonists (20). In addition, COX-2 expression is tightly associated with lipolysis, a cellular process controlled by adrenergic signaling in adipocytes (18, 21, 42). In the present study, we found that COX-2 expression in AT is suppressed by obesity in humans and in rodents and is restored by alternate-day fasting (Figure 1). In support of this, IF promotes production and release of COX-2 PG products from adipocytes, including PGE_2_ and PGI_2_ (Figure 2, D and E). As a result, COX-2 senses fasting and overfeeding in adipose tissue and serves a key player of adipose tissue microenvironment (Figures 4 and 5).

The beneficial effects of IF on insulin sensitivity and antiinflammation were diminished in mice lacking COX-2 in adipocytes, which is likely independent of an antiobesity effect (Figure 4), indicating that adipocyte COX-2 senses fasting and refeeding and mediates the beneficial effects of IF. Consistent with previous observations in adipocyte-specific COX-2–overexpressed mice (43), we also observed the antiobesity property of COX-2 in the present study (Figure 4); however, the underlying mechanisms are currently unclear. We also observed that COX-2 was expressed in AT of female rodents and its expression levels also were suppressed by obesity in humans (Figure 1). Whether COX-2 exerts an antiobesity effect in female rodents and humans remains to be addressed.

COX-2 promotes energy metabolism, such as beige adipocyte formation (18, 26, 44–46). PGE_2_, PGI_2_, or PGD_2_ induces beige adipogenesis yet has no significant effect on the activation of differentiated beige adipocytes (26), suggesting that PG promotes progenitor cell commitment to beige adipogenesis rather than activation of adipocytes. IF has been linked to beige adipogenesis in previous studies (3). However, it is unclear whether adipocyte COX-2 contributes to IF-induced white fat browning. COX-2 deficiency suppresses thermogenic gene expression under IF conditions with little effect under normal diet conditions (Supplemental Figure 3I). The remarkable induction of adipose COX-2 in IF-treated mice may explain this difference. In support of this, mTORC1 inactivation induces COX-2 expression and promotes beige adipocyte formation (26). Of note, adipocyte COX-2 only contributes 20%–30% of local PG production within AT (Figure 2, D and E). The contribution of adipocytes to the local PGs was significantly enhanced during fasting or starvation (Figure 2, D and E). Other metabolites, including fermentation products acetate and lactate and cytokines such as VEGF, mediate IF-elicited thermogenesis by targeting beige adipocytes and resident macrophages, respectively (3, 4). Our study suggests a new mechanism underlying IF-induced improvement in AT inflammation.

Although all 3 PGs (i.e., PGE_2_, PGD_2_, and PGI_2_) can facilitate beige adipocyte differentiation (26), only PGE_2_ exerts Treg-inducing effects by regulating its proliferation in white fat (Figure 6). This observation is supported by the recent finding that enrichment of PGE_2_ favors the immune-suppressive effects of adipose resident Tregs through its derived metabolite 15-keto-PGE_2_ (47). In agreement with the antiinflammatory action of PGE_2_ (17, 23, 48), we found that PGE_2_ promotes Treg proliferation but not differentiation within AT. Our results are consistent with the findings of Sharma, Baratelli, and colleagues (49) that PGE_2_ induces Foxp3 expression and Treg function in human lymphocytes and in lung cancer. In addition, AT Tregs use PGE_2_ as a substrate to generate PPARγ ligand 15-keto-PGE_2_ with hydroxyprostaglandin dehydrogenase (HPGD) to maintain AT homeostasis (47). Consistent with this, HPGD expression in Tregs was suppressed by the medium from COX-2–KO adipocytes, compared with HPGD expression in the control sample (Figure 3M), suggesting that adipocytes may be an important source of PGE_2_ that promotes the immune suppressive activity of Tregs. It is unaddressed whether PGE_2_ activates CD206^+^ macrophages and decreases γδT cell populations through regulation of Tregs. Given that Tregs promote the polarization of macrophages to an M2 phenotype by releasing cytokine IL-10 (50, 51), it is possible that PGE_2_ via Tregs alternatively activates CD206^+^ macrophages.

Notably, the effects of PGE_2_ on Tregs are still under debate. Some studies have shown that PGE_2_ induces Foxp3^+^CD4^+^CD25^+^ regulatory T cells in lung and in vitro, inhibiting effector T cell responses (52–54). In human lymphocytes, PGE_2_ appears to induce Foxp3 expression and Treg function (49). However, PGE_2_ was also reported to inhibit Treg differentiation via the EP2-cAMP/PKA signaling pathway (55). Consistent with the mediatory role of cAMP/PKA pathway in PGE_2_ action on T cells (56, 57), we observed that PGE_2_ treatment activated PKA and subsequently stimulated CaMKII activation and Treg proliferation (Figure 6F). Consistent with this, CaMKII-KO Tregs had an impaired response to PGE_2_ in terms of proliferation (Figure 6, G and H). The communication between adipocytes and Tregs is crucial for Treg proliferation, which also explains the relatively high percentage of Tregs (24%) in total CD4^+^ T cells in AT (Figure 3C) that we observed. Downregulation of COX-2 may be responsible for the decrease in the Treg fraction and subsequent adipose inflammation in obesity (Figure 4D). In contrast, activating the *Ptgs2* gene in adipocytes provides an approach to restoring AT Tregs and improving AT inflammation.

IF is suggested to bean effective way to improve adipose function and glucose homeostasis through targeting COX-2 and elevating PGE_2_ levels in AT (Figure 4D). On the other hand, while acute PGE_2_ levels have antiobesity and antiinflammatory effects in AT, whether chronic treatment of PGE_2_ potentially causes unwanted effects such as fever, increased metabolic rate, changes in activity level, systemic inflammation or pain, or reduced appetite and food intake necessitates further clarification.

In summary, our data show for the first time, to our knowledge, that adipocyte-derived PGE_2_ serves as a paracrine signal that promotes resident Treg proliferation by activating the PKA/CaMKII pathway. In addition, adipocyte-derived PGE_2_ mimics IF to elicit metabolic benefits, including Treg proliferation, type 2 inflammation, and insulin sensitivity. Our study shows that the COX-2/PGE_2_ axis in adipocytes is a checkpoint of AT inflammation in obesity. Moreover, this work provides critical insight into the mechanisms by which lipokines and immune cell populations contribute to IF-related mediation of obesity-related illness.

## Methods

### Materials.

Antibodies (antibody number) against the following were obtained from Cell Signaling Technology: COX-1 (4841), COX-2 (12282), PPARγ (2443), ERK p44/42 (9102), p-ERK P44/42 (4377), Akt (9272), p-Akt (T308) (9275), p-Akt (S473) (4051), and PKA (9621). CaMKII (sc-5306) and p-CaMKII (sc-32289) were from Santa Cruz Biotechnology. Polyclonal antibody to adiponectin and anti-tubulin were provided in-house as described previously (58). The anti-UCP1 (ab23841) was purchased from Abcam. PGD_2_, PGE_2_, and PGI_2_ were obtained from Cayman Chemical Company; KT 5720 from Sigma-Aldrich; and TATCN21 from Millipore.

### Human study.

Samples of visceral AT were obtained from 21 Chinese men and women. Ten participants had BMI >27 kg/m^2^ (obese/overweight group) and had undergone varicocelectomy or hysterectomy, and 11 had BMI <25 kg/m^2^ (control group) and had undergone varicocelectomy, salpingostomy, laparotomy, hysterectomy, debulking surgery, or myomectomy at Baodi Clinical College, Tianjin Medical University, Tianjin, China. Patients with cancer and other chronic inflammatory conditions were excluded. Patients’ baseline characteristics, including sex, age, BMI, BW, waist circumference, hip circumference, medications, and other comorbidities, are summarized in Supplemental Table 1.

The visceral ATs samples were obtained at the site of incision in the abdomen. Tissue specimens were immediately washed in saline buffer, snap frozen in liquid nitrogen and stored at –80°C until use. The tissue samples from visceral fat were used for Western blot analysis and RT-PCR. PCR primers are listed in the Supplemental Table 2.

### Animals.

The COX-2–floxed mice were provided by Harvey R. Herschman at the University of California at Los Angeles. Adipocyte–COX-2–specific KO mice were generated by crossing COX-2–floxed mice with adiponectin cre mice (Jackson Laboratory, catalog 10803). COX-2–floxed littermates were used as controls. The KO efficiency was confirmed in AT and other tissues by Western blot analysis and RT-PCR. We purchased C57BL/6 mice from Jackson Laboratory (catalog 000664). Unless otherwise noted, male mice were used for all experiments. Foxp3-eGFP mice (Jackson Laboratory, catalog 006772) were provided by Sarah Adams at the University of New Mexico Health Sciences Center (UNMHSC), and CaMKII-KO mice were originally from Wendong Huang’s laboratory at the City of Hope. Animals were housed in a specific pathogen-free barrier facility with a 12-hour light/12-hour dark cycle with free access to food and water. For the HFD challenge study, 6-week-old mice were fed a NCD provided by the animal facility at UNMHSC or a HFD (45% kcal fat) from Research Diets Inc. (catalog D124510) for 8 or 16 weeks, unless otherwise specified.

### Intermittent fasting.

We fed 6-week-old male COX-2–KO and control mice a NCD or HFD for 8 weeks and then randomly grouped them to either the Ad group or IF group. The Ad mice were allowed unrestricted access to their assigned diet, whereas the IF group was fasted for 24 hours and then fed during alternating 24-hour periods of free access to food. The alternate-day fasting was repeated for 3 or 15 cycles. Mice in the IF group were started with 24-hour fasting (10 am) followed by a 24-hour refeeding. The mice in the IF group were euthanized at the end of refeeding (10 am) for tissue sample collection after certain cycles of fasting and refeeding. Similar to the IF group, Ad mice were transferred into a new cage at 10 am every day. The tissue samples were collected during the light cycle (10 am) for both IF and Ad groups.

### Measurement of PG release.

To measure PG levels in tissue explants, 3-month-old male mice underwent 3 cycles of IF or Ad, and 2 types of fat pads, including eWAT and iWAT, were harvested and dissected. A tissue sample (~50 mg) was plated in 0.5 mL of medium (CaCl_2_, 1.28 mM; MgSO_4_, 1.2 mM; NaHCO_3_, 25 mM; HEPES, 15 mM; BSA 0.5%; d-glucose, 5 mM; and penicillin/streptomycin 1%) and incubated at 37°C, 5% CO_2_ for 8 hours. Media were then collected and centrifuged at 2300*g* for 5 minutes at 4°C, and the supernatant was snap frozen. The concentration of PGs, including PGE_2_, PGD_2_, and PGI_2_, in the media was determined using enzyme immunoassay kits according to the manufacturer’s protocol (Cayman Chemical), as described in our previous study (26). The secretion of PGE_2_, PGD_2_, and PGI_2_ was normalized using wet tissue mass. To measure the levels of secreted PGs in primary adipocytes, preadipocytes from iWAT were cultured and differentiated into adipocytes, as described previously (59). Differentiated adipocytes were starved for 4 hours in 0.4% BSA/DMEM, washed with PBS, and incubated with fresh DMEM containing 1% BSA for 2 hours. Culture medium was collected and used to determine the secretion levels of PGE_2_, PGD_2_, and PGI_2_ that were normalized by the total protein level of cells.

### Administration of PGE_2_ or anti-CD25.

PGE_2_ was dissolved in 100% DMSO and diluted in 0.9% sodium chloride to reach the concentration of 5 μg/mL. We fed 8-week old COX-2–KO mice an HFD for 8 weeks and then randomly grouped the mice into Ad and IF groups. Two weeks after IF, mice were administered 50 μg/kg PGE_2_ via i.p. injection every other day for 2 weeks. For the neutralization of CD25 to block Treg function, 8-week-old C57BL/6 mice were fed an HFD for 8 weeks and then administered neutralizing antibody anti-mouse CD25 (IL-2Ra) or IgG1 isotype control anti-trinitrophenol (Bio X Cell) via i.p. injection every other day for 2 weeks starting 2 days before PGE_2_ treatment. The mice were then euthanized, and tissue samples from epididymal and inguinal fat were collected for flow cytometry and RT-PCR analyses.

### Primary culture and differentiation of adipocytes.

Primary stromal vascular fractions (SVFs) from iWAT of 3-week-old COX-2–KO and control mice were isolated, cultured, and differentiated into adipocytes according to the procedure described previously (59). Day-6 differentiated primary COX-2–KO and control adipocytes were cultured with a fresh differentiation medium for 24 hours. The medium was collected and used for the determination of PG levels. In addition, the medium was also used to treat primary Tregs isolated from AT (see *Treatment of primary Tregs*).

### Flow cytometry.

The suspended SVFs from adipose depots were fixed, Fc blocked, and stained with conjugated antibodies, including anti-CD45 (Biolegend), anti-Siglec-5 (BD Pharmingen), anti-CD11b (eBioscience), and anti-CD206 (eBioscience) to identify macrophage and eosinophil subsets. The Treg populations were stained with conjugated antibodies, including anti-CD4 and anti-Foxp3 (eBioscience). Ki67 (eBioscience) was used to mark the proliferating cells. The γδT cell populations were Fc blocked and labeled with antibodies to peridinin chlorophyll protein complex–conjugated anti-CD3 (Biolegend), and antigen-presenting cell–conjugated anti-γδT cell receptor (eBioscience) by fixation. Flow cytometry analysis was performed on an acoustic focusing cytometer Attune NxT (Invitrogen), and the data were analyzed by FlowJo software as described in our previous studies (2, 60).

### Adoptive Treg transfer.

Foxp3-eGFP mice were used as the donor mice for immune cell transfer experiments (47, 61). We administered 50 μg of Treg-Protector (Biolegend) through i.v. injection into the donor mice 30 minutes before harvesting lymphoid organs, to protect against cell death. Mouse CD4^+^CD25^+^ Tregs were isolated from a single-cell suspension of lymph nodes and spleens by magnetic-activated cell sorting using a CD4^+^CD25^+^ Regulatory T Cell Isolation Kit (Miltenyi Biotec) with a 2-step procedure: positive selection of CD4^+^CD25^+^ cells after negative depletion of non-CD4^+^ cells, as described previously (62). The CD4^+^GFP^+^ cells were selected from the isolated CD4^+^CD25^+^ cells and i.p. injected (1 × 10^5^ cells/mouse) into male COX-2–KO and control mice fed an HFD for 8 weeks. At 14 days after injection, the recipients underwent glucose and insulin tolerance tests, as described previously (63). BW was measured as described in Results. The CD4^+^Foxp3 (GFP)^+^ Tregs and γδT cells in AT were analyzed using flow cytometry.

### Treatment of primary Tregs.

For the treatment of AT Tregs, CD4^+^CD25^+^ T cells were isolated from eWAT stromal vascular fraction of 2-month-old mice by magnetic-activated cell sorting as described in the previous paragraph. Isolated CD4^+^CD25^+^ cells were cultured for 24 hours, followed by treatment with a different-dose of PG for another 24 hours. For the inhibitor experiment, the cells were pretreated with 5 μM KT 5720, a specific inhibitor of PKA, or 5 μM TNTCN21, a specific inhibitor of CaMKII, for 1 hour prior to co-treatment with 100 nM PGE_2_. For CaMKII phosphorylation study, cells were treated with or without 100 nM PGE_2_ for 1 hour before flow cytometry analysis. In the treatment of AT Tregs with the medium from adipocytes, the medium was collected as described for primary culture and then used to treat AT Tregs for 2 days. After treatment, Tregs underwent flow cytometry analysis. For the signaling study**, **naive CD4^+^ T cells were isolated from a single-cell suspension of lymph nodes and spleens by depleting non-CD4^+^ and regulatory T cells (B220^+^, CD8^+^, and CD25^+^ cells) and positive selecting CD62L^+^ cells using magnetic beads. Naive T cells were cultured in 10% FBS-PRMI 1640 medium in the presence of 2 ng/mL TGF-β, 10 ng/mL IL-2, and 5 μg/mL anti–IFN-γ on anti–CD3/CD28–coated 24-well plates for 4 days. One day after differentiation, the differentiated Tregs were treated with or without PGE_2_ with different doses. Cells were harvested for Western blot analysis.

H&E staining, liver triglyceride content, real-time PCR, Western blot, glucose and insulin tolerance test and insulin signaling assay, and dual-energy X-ray absorptiometry scanning are detailed in the Supplemental Methods. See complete unedited blots in the supplemental material.

### Statistics.

Statistical analysis of the data was performed using a 2-tailed Student’s *t* test between 2 groups or the 1-way ANOVA for a comparison of more than 2 groups. All the results are presented as the mean ± SEM, and *P* < 0.05 was considered statistically significant.

### Study approval.

All animal experimental protocols were reviewed and approved by the Animal Care Committee of the University of New Mexico Health Sciences Center. The human study complied with the Helsinki Declaration for investigation of human participants and received ethical approval from the IRBs of Baodi Clinical College, Tianjin Medical University. All participants provided written informed consent.

## Author contributions

ML designed the project. XOY designed the adoptive transfer of Tregs and provided technical support for the Treg analysis for this project. FL served as a consultant on this project. LL, XM, CY, and YF completed the human study. CW, XZ, LL, YL, XY, XD, HL, LW, LERF, and LY conducted the experiments. CW, XZ, YL, HL, XD, WH, and ML analyzed the results. CW drafted the Methods section. ML is the guarantor of this study and wrote the manuscript. FL, XOY, and LERF edited the manuscript. All authors reviewed and approved the manuscript.

## Supplementary Material

Supplemental data

## Version 1 03/08/2022

Electronic publication
