# Supplementary material for: Adipocyte-derived PGE2 is required for intermittent fasting–induced Treg proliferation and improvement of insulin sensitivity
Source: JCI Insight. 2022 Mar 8;7(5):e153755. doi: 10.1172/jci.insight.153755 (PMC8983131; doi:10.1172/jci.insight.153755)
Supplement: Supplemental data [file jciinsight-7-153755-s219.pdf]

Supplementary information

Methods

H&E staining For the H&E staining, tissue samples were fixed with 4% formalin for 48 hrs and embedded in paraffin. Four sections (6-μm thick) per tissue were stained with H&E according to the standard protocols and analyzed using the NIH Image J software. To analyze the adipocyte size in adipose tissue, stained tissue sample were visualized with NanoZoomer Slide Scanner. Eight representative images per section were used to determine adipocyte size. Size of adipocytes were determined with ImageJ Analysis as we described previously (*1*).

Liver triglyceride content 100-300 mg of frozen liver tissue samples were digested using 350 μl potassium hydroxide (ethanol:30% potassium hydroxide=2:1) at 55℃ overnight. Brought volume to 1,000 μl with H_2O: ethanol (1:1), then spined in a microfuge for 5 minutes to get the supernatant. 200 μl supernatant was mixed with 215 μl 1 M MgCl2 and chilled on ice for 10 minutes. After centrifugation, the supernatant was used for the measurement of TG content reflected by the levels of Free Glycerol Reagent (Sigma-Aldrich, F6428) according to the protocol.

Real-time PCR Total RNA was extracted from tissue samples using the RNeasy Lipid Tissue Mini Kit (Qiagen). The purity and concentration of total RNA were determined by a Nano Drop spectrophotometer (Thermo Fisher). 1 μg of total RNA was reverse-transcribed using cDNA kit (AB Applied Biosystems). Real-time PCR amplification was detected using SYBR Green PCR master mixture (Qiagen) on a Roche 480 Real-time PCR system. Primer sequences are listed in the Supplemental Table 2. Each pair of primers amplified products spanning several exons, distinguishing spliced mRNA from genomic DNA contamination. The reaction yielded about 200 bp of PCR products for specified genes and internal control genes. The relative expression levels of target genes were normalized to *tubulin*.

Western blot For tissue samples, frozen samples of brown adipose tissue were sliced and about 1 mg of tissue sample was homogenized for Western blot analysis. The protein in the homogenate was normalized and separated with SDS gel followed with immunoblots as described previously (*2*). For the iWAT samples, tissue samples around the lymph node in the inguinal fat was used for the Western blot considering given the heterogeneity of iWAT in cell type (*2*). Cell lysates were prepared in ice-cold lysis buffer and the general procedures were used for Western blot as described in our previous study (*3*). For all the Western blot data presented in this study, the X-axis indicates the individual mouse or the individual cell treatment. When multiple target proteins had similar molecular weight or were unable to be obtained individually from the same gel, the gels were run in parallel with the same order and the same individual volume of samples. Quantification of protein levels was performed by analyzing Western blots using the Scion Image Alpha 4.0.3.2 program (Scion Corp.). The expression level for a particular protein was normalized with the internal control protein and the relative change in protein level was expressed as percentage of control protein levels. The relative level of each control sample was arbitrarily set at 1.0 in the cell and in vivo studies.

Glucose/insulin tolerance test and insulin signaling assay Mice were fasted for 16 hrs, and blood glucose levels were measured using a Glucometer (GE Diabetes, USA) by tail bleeds. Mice were then intraperitoneally injected with 2 g glucose/kg of body mass, and blood glucose levels were measured at the time of 15 min, 30 min, 60 min and 120 min post glucose injection. For insulin tolerance test, mice were fasted for 4 hrs, and blood glucose levels were measured. Mice were then

intraperitoneally injected with 0.75 U/mL insulin/kg of body weight, and blood glucose levels were measured at the time of 15 min, 30 min, 60 min and 90 min post insulin injection. For the assay of insulin signaling, mice were fasted overnight and administered with 5U/kg bodyweight insulin for 5 min, following with tissue sample collection and Western blot as described previously (*4*).

DEXA scanning To check the body composition, 3 month or 6 month-old mice were anesthetized through intraperitoneal injection with 0.1 mL/10 grams animal body weight Ketamine/Xylazine (10 mg/mL Ketamine and 1 mg/mL Xylazine). Bone mineral density, lean mass, fat mass, total body weight and fat percentage were determined by dual-energy X-ray absorptiometry (DEXA) (GE Medical Systems, Madison, WI) as we described previously (*1*).

Table 1 Baseline patient characteristics

Gender	Age	Height (CM)	Body weight (Kg)	Waist circumference (CM)	Hip circumference (CM)	BMI	Illness	Surgery	Fasting serum glucose (UM)	Hypertension (Y/N)	Diabetes (Y/N)	Coronary artery disease (Y/N)	Smoke (Y/N)	Sleep Apnea (Y/N)	Medicine
Male	19	180	59	71	89	18.2	Varicocele	Varicocelectomy	5.3	N	N	N	N	N	N
Female	48	156	60	79	98	24.65	Adenomyosis	Laparotomy	4.67	N	N	N	N	N	N
Female	44	148	49	76.5	91	22.37	Adenomyosis	Laparotomy	4.53	N	N	N	N	N	N
Female	32	157	52	74	96	21.09	Ovarian Cancer	Hysterectomy	4.45	N	N	N	N	N	N
Female	55	163	63	86	102	23.71	Ovarian Cancer	debulking surgery	5.02	N	N	N	N	N	N
Female	38	168	68	85	94.5	24.09	Leiomyoma	Myomectomy	4.55	Y	Y	N	N	N	Statin
Female	31	170	69	82	100	23.87	Leiomyoma	Myomectomy	4.67	N	N	N	N	N	N
Male	31	190	75			20.78	Varicocele	Varicocelectomy	6.54	N	N	N	N	N	N
Male	31	165	66			24.24	Right inguinal hernia	Laparoscopic surgery	5.03	N	N	N	N	N	N
Male	64	174	75			24.77	Right inguinal hernia	Laparoscopic surgery	5.92	N	N	N	N	Y	Telmisartan, amlodipine
Male	14	176	75			24.21	Varicocele	Varicocelectomy	6.11	N	N	N	N	Y	N
Female	50	170	87	93	110	30.10	Leiomyoma	Hysterectomy	4.59	N	N	N	N	N	N
Female	45	159	77	92	108	30.5	Leiomyoma	Hysterectomy	4.91	N	N	N	N	N	N
Female	29	159	78	91	110	30.85	Ovarian Cancer	Hysterectomy	4.13	N	N	N	N	Y	N
Male	23	175	95	99	108	31	Varicocele	Varicocelectomy	4.95	N	N	N	N	Y	N
Female	29	170	99	93	109	34.25	Ovarian Cancer	Hysterectomy	5.22	N	N	N	N	N	N
Female	35	160	80	95	116	31.25	Ovarian Cancer	Hysterectomy	5.36	N	N	N	N	N	N
Female	35	162	92	96	111	35.06	Ectopic pregnancy	Salpingostomy	4.82	N	N	Y	N	N	Aspirin
Female	33	158	85	97	106	34	Pelvic mass	Hysterectomy	4.16	N	N	N	N	Y	N
Male	59	170	89			30.80	Abdominal wall hernia	Laparoscopic ventral hernia repair	4.96	N	N	N	Y	N	N
Male	52	177	85	97	105	27.13	Varicocele	Varicocelectomy	5.32	N	N	Y	N	Y	N

Table 2. List of primer sequences used in real-time PCR.

Gene	Forward sequence (5’ to 3’)	Reverse sequence (5’ to 3’)
TUBULIN	CTGGACCGCATCTCTGTGTACT	GCCAAAAGGACCTGAGCGAACA
PTGS2	CGGTGAAACTCTGGCTAGACAG	GCAAACCGTAGATGCTCAGGGA
PTGS1	GATGAGCAGCTTTTCCAGACGAC	AACTGGACACCGAACAGCAGCT
Tubulin	CAGGCCGGACAGTGTGGCAAC	GGCTTCATTATAGTACACAGAGATTCG
Ptgs2	CTCACGAAGGAACTCAGCACT	TAGAATCCAGTCCGGGTACAGT
Ptgs1	GTGCTGGGGCAGTGCTGGAG	TGGGGCCTGAGTAGCCCGTG
Foxp3	CACCTATGCCACCCTTATCCG	CATGCGAGTAAACCAATGGTAGA

Gata3	TTTACCCTCCGGCTTCATCCTCCT	TGCACCTGATACTTGAGGCACTCT
Il10	CTTACTGACTGGCATGAGGATCA	GCAGCTCTAGGAGCATGTGG
Tgfb1	CCACCTGCAAGACCATCGAC	CTGGCGAGCCTTAGTTTGGAC
Tgfb2	CTTCGACGTGACAGACGCT	GCAGGGGCAGTGTAAACTTATT
Tgfb3	CCTGGCCCTGCTGAACTTG	TTGATGTGGCCGAAGTCCAAC
Il1b	GAAATGCCACCTTTTGACAGTG	TGGATGCTCTCATCAGGACAG
Il6	CTGCAAGAGACTTCCATCCAG	AGTGGTATAGACAGGTCTGTTGG
Tnf	CAGGCGGTGCCTATGTCTC	CGATCACCCCGAAGTTCAGTAG
Ifng	ATCTGGAGGAACTGGCAAAA	TTCAAGACTTCAAAGAGTCTGAGGTA
Fgf21	AGATCAGGGAGGATGGAACA	TCAAAGTGAGGCGATCCATA
Vegfa	GCACATAGAGAGAATGAGCTTCC	CTCCGCTCTGAACAAGGCT
H2-Eb1	GCGGAGAGTTGAGCCTACG	CCAGGAGGTTGTGGTGTTCC
Hpgd	ATCGGATTCACACGCTCAG	TGGGCAAATGACATTCAGTC

References

1. Zhang X, et al. Adipose mTORC1 Suppresses Prostaglandin Signaling and Beige Adipogenesis via the CRTC2-COX-2 Pathway. *Cell Rep.* 2018*;* 24(2):3180-3193.
2. Liu M, et al. Fat-specific DsbA-L overexpression promotes adiponectin multimerization and protects mice from diet-induced obesity and insulin resistance. *Diabetes.* 2012*;*61(11):2776-2786.
3. Chi J, et al. Three-Dimensional Adipose Tissue Imaging Reveals Regional Variation in Beige Fat Biogenesis and PRDM16-Dependent Sympathetic Neurite Density. *Cell Metab,* 2018;27:226-236.
4. Liu M, et al. A disulfide-bond A oxidoreductase-like protein (DsbA-L) regulates adiponectin multimerization. *Proc Natl Acad Sci U S A.* 2008;105(47):18302-18307.

Figure legends

Fig. S1. Intermittent fasting induces COX-2 expression and adipocyte-specific depletion of COX-2 leads to adiposity and insulin resistance.

4-month-old C57BL/6 male mice processed with alternate day fasting for 6 days and the mice were used for the following studies from Figs. S1A to S1D. The expression levels of COX-2 but not COX-1 in protein (**A**) and mRNA (**B**) were induced by IF in eWAT. The levels of COX-2 and COX-1 in protein (**C**) and mRNA (**D**) in iWAT were not significantly induced by IF. **E**. Suppression of COX-2 by COX-2 knockdown in monocytes and COX-2 KO in adipocytes. The expression of COX-1 and COX-2 in eWAT (**F**) and iWAT (**G**) of COX-2 KO and control mice treated with or without IF. **H**. COX-2 KO slightly increased PGD2 levels in eWAT but not in iWAT under Ad conditions, while IF had no significant effect on PGD2 levels in both eWAT and iWAT. **I**. Body mass and fat mass were slightly increased in 3-month-old COX-2 KO

compared to control mice, although there was no significant difference in fat percentage. Lean mass, fat mass, total body mass and fat percentage were determined using DEXA Scanning. The glucose tolerance (**J**) and insulin tolerance (**K**) were slightly decreased in 6-month-old COX-2 KO mice compared to control mice with lesser extent at 3-month age (**L** and **M**). Data are presented as the mean±SEM. *P<0.05 or **P<0.01. A t-test was used for statistical analysis for Figs. S1A to S1D and S1H-I. ANOVA was used for statistical analysis in Figs. S1J-M.

Fig. S2 Deficiency of COX-2 in adipocytes alters immune response in adipose tissue.

A. Strategies of flow cytometry analysis to examine different populations of immune cell types in WAT. Adipose tissue samples from 6-month-old male COX-2 KO and control mice were collected, and SVFs of eWAT were used for Flow Cytometry analysis. There was also no significant difference in fraction of resident $CD4^+$ (**B**), $CD3^+$ (**C**) and $CD8^+$ (**D**) cell cells between COX-2 KO and control mice. **E**. The fraction of resident $CD45^+$ cell was decreased by COX-2 KO in eWAT, although the difference did not reach the significance. COX-2 KO mice displayed reduced $CD11b^+$ population (in total SVFs) (**F**) but increased ratio of $CD206^-$ cells (M1) in total $CD11b^+$ cells in eWAT (**G**). **H**. The percentages of $Siglec5^+$ cells in total $CD45^+$ cells were decreased in eWAT. Data are presented as the mean±SEM. *P<0.05 or **P<0.01. A t-test was used for the statistical analysis in Figs. S2B to S2H.

Fig. S3 COX-2 deficiency in adipocyte abrogates the improving effect of IF on hepatic steatosis, while has no significant effect on the fat loss.

A. H&E staining of eWAT, iWAT, BAT and liver in HFD-fed COX-2 KO and control mice with or without intermittent fasting. **B**. Quantification of adipocyte size in eWAT and iWAT in Figs. S3A. * or ** for the comparison between Ctrl Ad and KO Ad. # or ## for the comparison between Ctrl IF and KO IF. COX-2 depletion did not significantly affect the body mass losing (**C**), the food intake of each cycle during IF (**D**), while suppressed hepatic TG (**E**) reducing effects of IF. *P<0.05, Ctrl vs KO with Ad diet, $^{\#}$P<0.05, Ad vs IF in control mice; $^{\$}$P<0.05, Ad vs IF in KO mice. COX-2 deficiency diminished intermittent fasting-mediated increase in $CD11b^+CD206^+$ population (**F**) and the ratio of $CD11b^+CD206^+$ in $CD11b^+$ cells (**G**) in eWAT. **H**. Insulin-stimulated phosphorylation of Akt at Thr308 and Ser473 in eWAT of COX-2 KO and control mice treated with or without IF. n=4/group. **I**. Intermittent fasting-induced thermogenic gene expression was suppressed by COX-2 deficiency in iWAT. In addition, COX-2 deficiency suppressed adiponectin expression and induced PPARγ in iWAT which was not significantly affected by intermittent fasting. **J**. The mRNA levels of FGF21 and VEGF in iWAT and IFNg and MHCII (H2-eb1) in eWAT under Ad and IF feeding conditions. **K**. COX-2 KO had no significant effect on the secretion levels of PGD2 in white adipocytes. PGD2 levels were determined by ELISA kit. **L**. Treatment of PGE2 had little effect on the phosphorylation of Akt and Erkp44/42 in differentiated Tregs. $CD4^+$ Naive T cells were isolated from a single-cell suspension from lymph nodes and spleens and then differentiated into $CD4^+Foxp3^+$ Treg cells. Differentiated Tregs were treated with or without PGE2 for 1 hr. **M**. Inhibiting PKA by 5 μM KT5720 or Inhibiting CaMKII by 5 μM TATCN21 suppressed PGE2 treatment-induced phosphorylation of CaMKII in differentiated Tregs. Tregs were treated with KT5720 or TATCN21 for 1 hour followed with co-treatment with PGE2 for another hour. n=3/group. **N**. The expression and phosphorylation of CaMKII were markedly suppressed by depletion of CaMKII in BAT, iWAT and skeletal muscle. Figs. S3B, S3C, S3E, S3F-S3J were analyzed using ANOVA, and S3K was analyzed using t-test. Data are presented as the mean±SEM, *P<0.05 or **P<0.01.

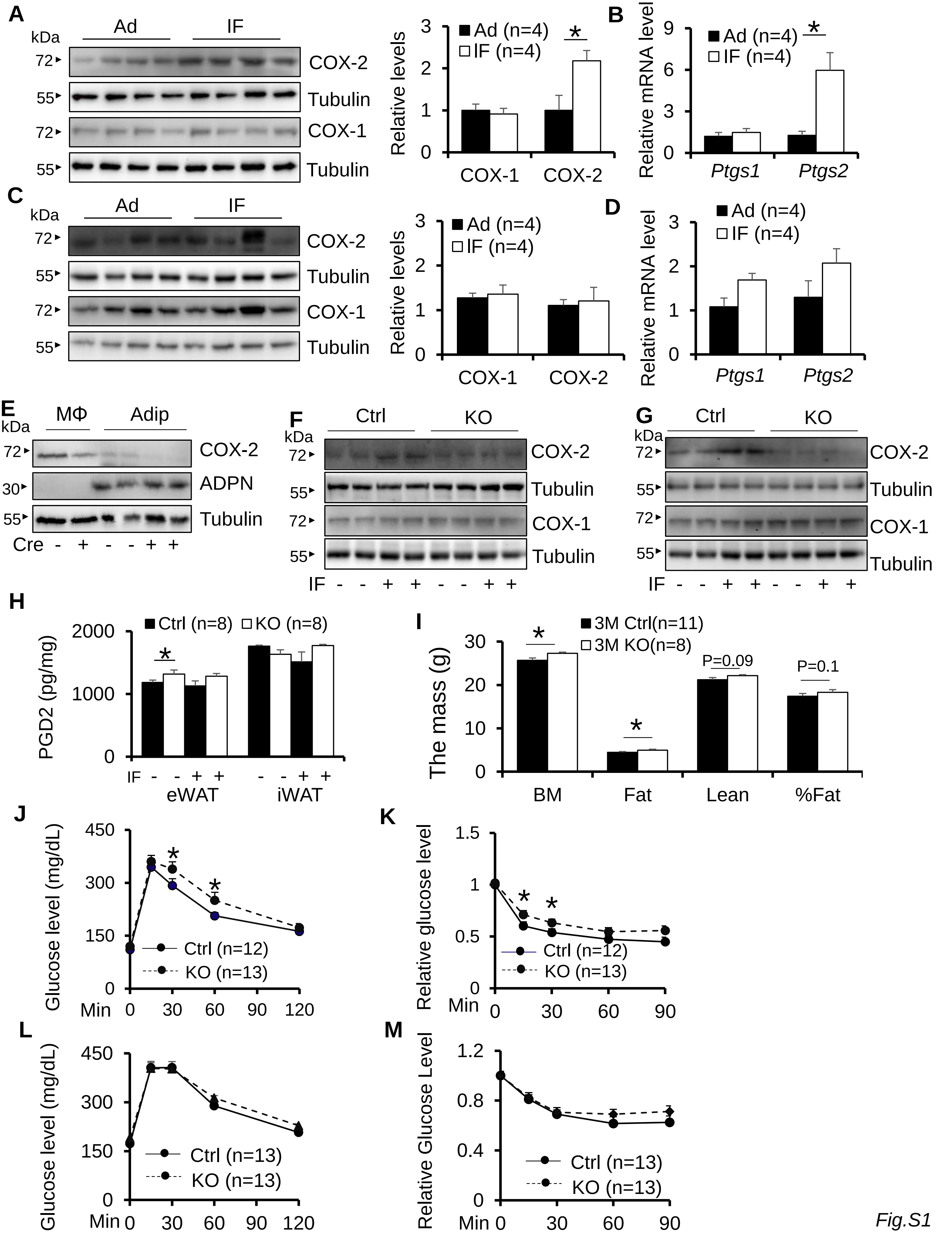
A
Ad
IF
kDa
COX-2
Tubulin
COX-1
Tubulin
Relative levels
Ad (n=4)
IF (n=4)
COX-1
COX-2
B
Relative mRNA level
Ptgs1
Ptgs2
C
D
E
MΦ
Adip
ADPN
Cre
F
Ctrl
KO
G
H
PGD2 (pg/mg)
Ctrl (n=8)
KO (n=8)
eWAT
iWAT
I
3M Ctrl(n=11)
3M KO(n=8)
The mass (g)
P=0.09
P=0.1
BM
Fat
Lean
%Fat
J
Glucose level (mg/dL)
Ctrl (n=12)
KO (n=13)
Min
K
Relative glucose level
L
Ctrl (n=13)
KO (n=13)
M
Relative Glucose Level
Fig.S1

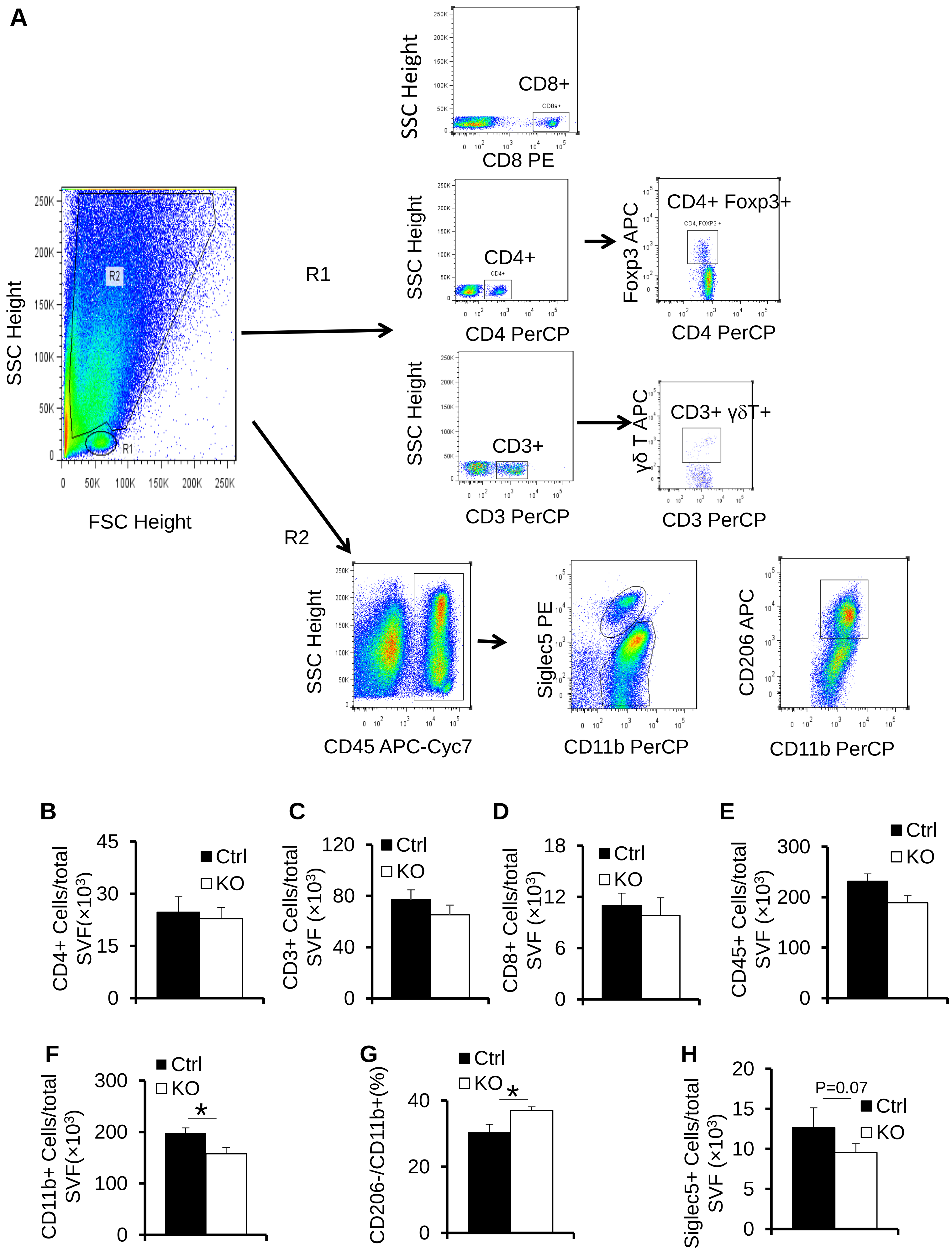

A
SSC Height
FSC Height
R2
R1
R1
R2
CD8+
CD8 PE
CD4+
CD4 PerCP
CD4+ Foxp3+
Foxp3 APC
CD3+
CD3 PerCP
CD3+ γδT+
γδ T APC
CD45 APC-Cyc7
Siglec5 PE
CD11b PerCP
CD206 APC
B
CD4+ Cells/total SVF(×10³)
C
CD3+ Cells/total SVF (×10³)
D
CD8+ Cells/total SVF (×10³)
E
CD45+ Cells/total SVF (×10³)
F
CD11b+ Cells/total SVF(×10³)
G
CD206-/CD11b+(%)
H
Siglec5+ Cells/total SVF (×10³)
P=0.07
Ctrl
KO
*

Fig.S2

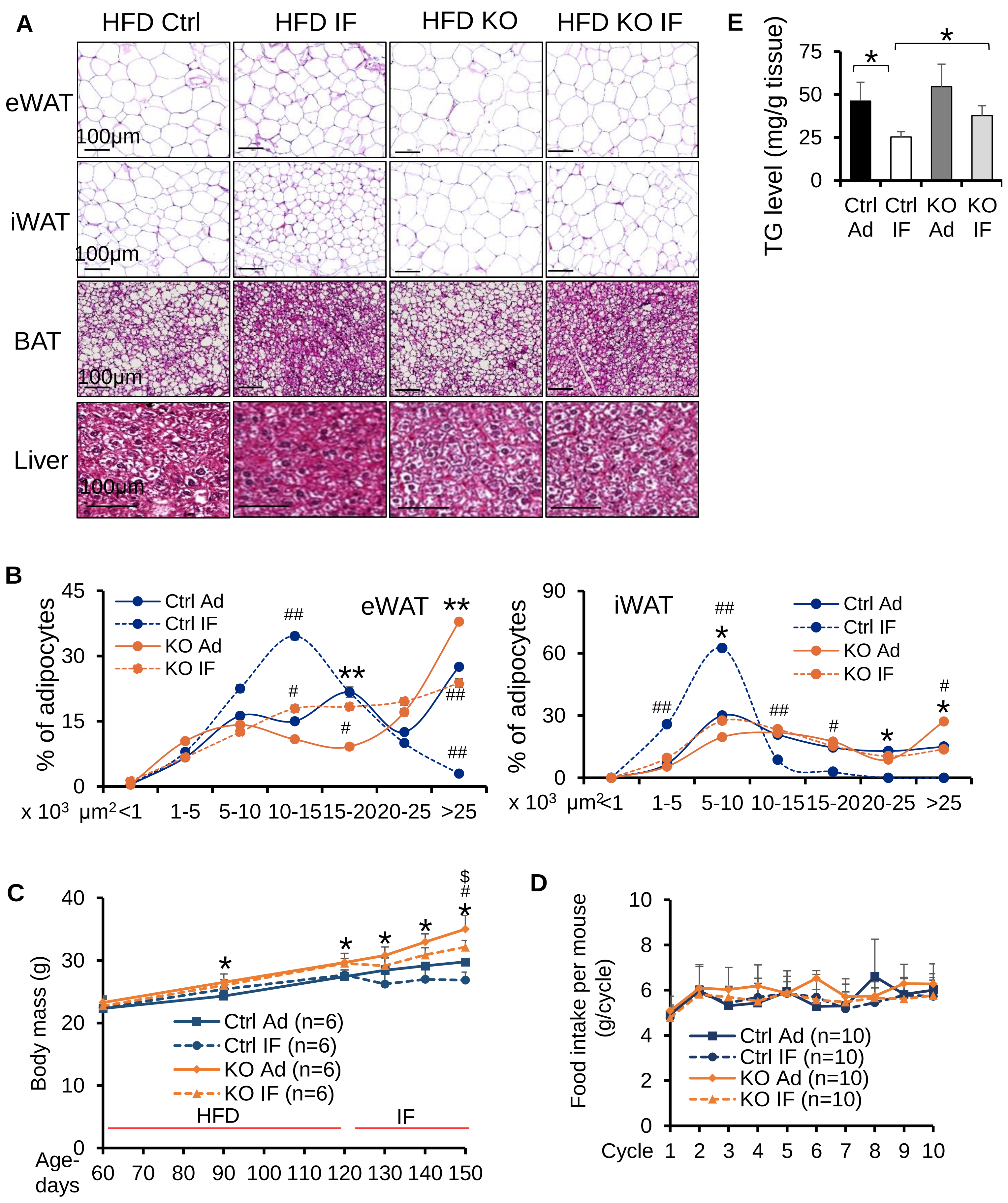
A
HFD Ctrl
HFD IF
HFD KO
HFD KO IF
eWAT
iWAT
BAT
Liver
100μm
E
TG level (mg/g tissue)
75
50
25
0
Ctrl Ad
Ctrl IF
KO Ad
KO IF
B
% of adipocytes
eWAT
iWAT
Ctrl Ad
Ctrl IF
KO Ad
KO IF
x 10³ μm² <1 1-5 5-10 10-15 15-20 20-25 >25
C
Body mass (g)
Ctrl Ad (n=6)
Ctrl IF (n=6)
KO Ad (n=6)
KO IF (n=6)
HFD
IF
Age-days 60 70 80 90 100 110 120 130 140 150
D
Food intake per mouse (g/cycle)
Ctrl Ad (n=10)
Ctrl IF (n=10)
KO Ad (n=10)
KO IF (n=10)
Cycle 1 2 3 4 5 6 7 8 9 10

Fig. S3

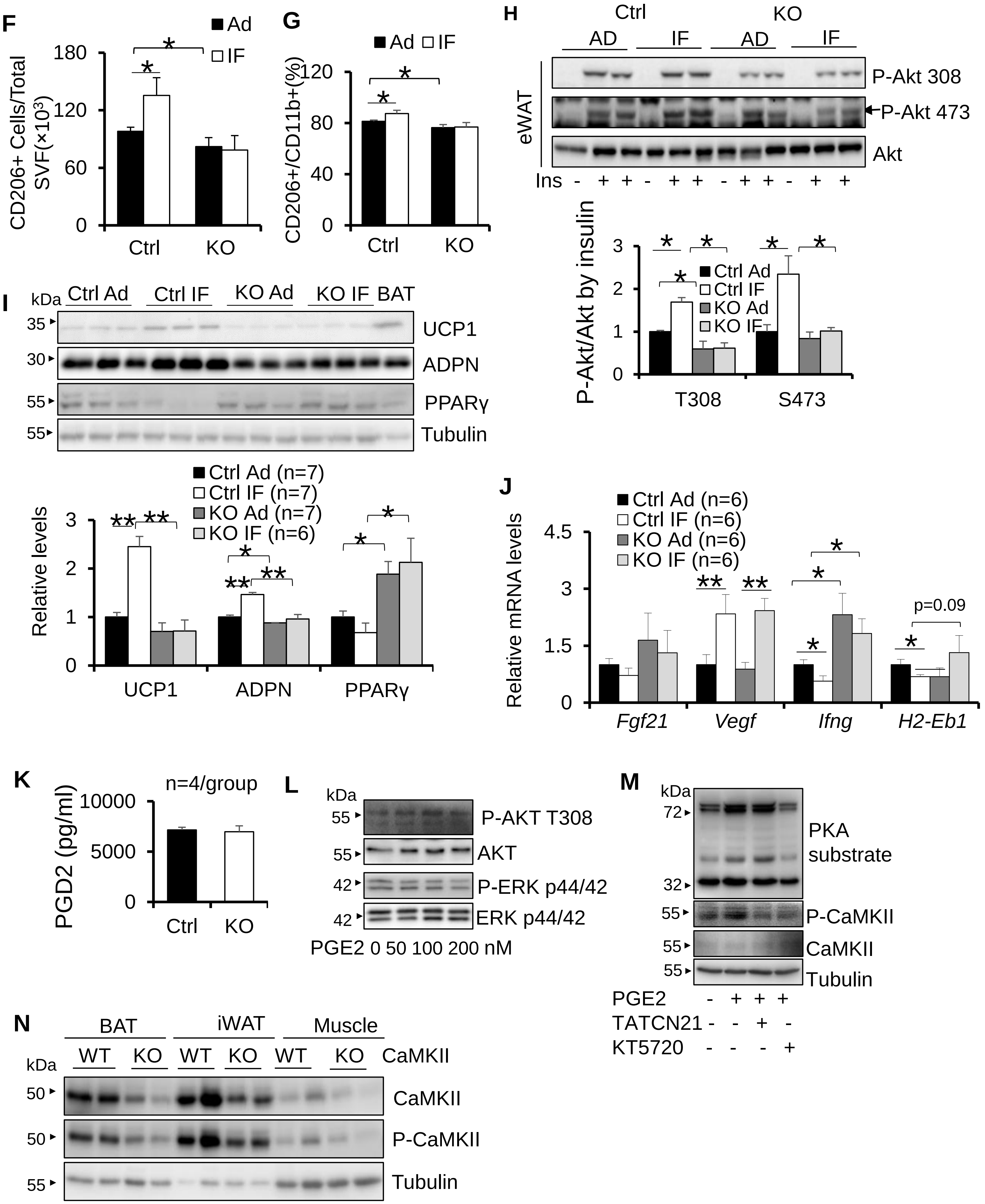

F
Ad
IF
CD206+ Cells/Total SVF(×10³)
180
120
60
0
Ctrl
KO
G
CD206+/CD11b+(%)
120
80
40
0
H
Ctrl
KO
AD
IF
eWAT
P-Akt 308
P-Akt 473
Akt
Ins
P-Akt/Akt by insulin
Ctrl Ad
Ctrl IF
KO Ad
KO IF
T308
S473
I
kDa
BAT
35
30
55
UCP1
ADPN
PPARγ
Tubulin
Ctrl Ad (n=7)
Ctrl IF (n=7)
KO Ad (n=7)
KO IF (n=6)
Relative levels
J
Ctrl Ad (n=6)
Ctrl IF (n=6)
KO Ad (n=6)
KO IF (n=6)
Relative mRNA levels
4.5
3
1.5
p=0.09
Fgf21
Vegf
Ifng
H2-Eb1
K
n=4/group
PGD2 (pg/ml)
10000
5000
L
P-AKT T308
AKT
P-ERK p44/42
ERK p44/42
PGE2 0 50 100 200 nM
M
72
32
PKA substrate
P-CaMKII
CaMKII
PGE2
TATCN21
KT5720
N
BAT
iWAT
Muscle
WT
KO
CaMKII
50

Fig. S3
